# Implications of Microorganisms in Alzheimer’s Disease

**DOI:** 10.3390/cimb44100314

**Published:** 2022-09-30

**Authors:** Pardeep Yadav, Yeon-Hee Lee, Hrithika Panday, Shubham Kant, Neha Bajwa, Ritika Parashar, Saurabh Kumar Jha, Niraj Kumar Jha, Parma Nand, Sang-Soo Lee, Abhimanyu Kumar Jha

**Affiliations:** 1 Department of Biotechnology, School of Engineering & Technology, Sharda University, Greater Noida 201310, Uttar Pradesh, India; 2 Institute for Skeletal Aging & Orthopedic Surgery, Hallym University-Chuncheon Sacred Heart Hospital, Chuncheon-si 24252, Gangwon-do, Korea; 3 Department of Pharmaceutical Sciences & Technology, Maharaja Ranjit Singh Punjab Technical University, Bathinda 151001, Punjab, India; 4 Department of Biotechnology Engineering and Food Technology, Chandigarh University, Mohali 140413, Punjab, India; 5 Department of Biotechnology, School of Applied and Life Sciences (SALS), Uttaranchal University, Dehradun 248007, Uttarakhand, India

**Keywords:** Alzheimer’s disease, neuroinflammation, neurodegeneration, inhibitors, gut microbiota, beta-secretase, gamma-secretase, blood–brain barrier

## Abstract

Alzheimer’s disease (AD) is a deadly brain degenerative disorder that leads to brain shrinkage and dementia. AD is manifested with hyperphosphorylated tau protein levels and amyloid beta (Aβ) peptide buildup in the hippocampus and cortex regions of the brain. The nervous tissue of AD patients also contains fungal proteins and DNA which are linked to bacterial infections, suggesting that polymicrobial infections also occur in the brains of those with AD. Both immunohistochemistry and next-generation sequencing (NGS) techniques were employed to assess fungal and bacterial infections in the brain tissue of AD patients and non-AD controls, with the most prevalent fungus genera detected in AD patients being Alternaria, Botrytis, Candida, and Malassezia. Interestingly, Fusarium was the most common genus detected in the control group. Both AD patients and controls were also detectable for Proteobacteria, followed by Firmicutes, Actinobacteria, and Bacteroides for bacterial infection. At the family level, *Burkholderiaceae* and *Staphylococcaceae* exhibited higher levels in the brains of those with AD than the brains of the control group. Accordingly, there is thought to be a viscous cycle of uncontrolled neuroinflammation and neurodegeneration in the brain, caused by agents such as the herpes simplex virus type 1 (HSV1), *Chlamydophila* *pneumonia*, and Spirochetes, and the presence of apolipoprotein E4 (APOE4), which is associated with an increased proinflammatory response in the immune system. Systemic proinflammatory cytokines are produced by microorganisms such as Cytomegalovirus, *Helicobacter pylori*, and those related to periodontal infections. These can then cross the blood–brain barrier (BBB) and lead to the onset of dementia. Here, we reviewed the relationship between the etiology of AD and microorganisms (such as bacterial pathogens, Herpesviridae viruses, and periodontal pathogens) according to the evidence available to understand the pathogenesis of AD. These findings might guide a targeted anti-inflammatory therapeutic approach to AD.

## 1. Introduction

Alzheimer’s disease (AD) is a deadly neurodegenerative illness that mostly impacts the elderly and is a major health concern for the geriatric population worldwide. AD prevalence increases substantially with age, reaching 50% in 85-year-olds [1]. AD is expected to become significantly more common in the geriatric population as the median life expectancy grows. The worldwide incidence of dementia is about 24 million, and it is estimated to surge fourfold by the year 2050 [2]. As such, there is a need for modern treatment options given the probable risk factors for AD and treatments that can delay AD onset and occurrence [3].

About 5000 species of gut microorganisms, notably Firmicutes, Bacteriodetes, Actinobacteria, and Proteobacteria, have been reported in the human intestinal lumen. These microbes play crucial roles in gut digestion and absorption functions [4]. Among these microorganisms, in particular, *Chlamydia pneumoniae* and Spirochetes have multiple research findings pointing to their significance in the pathogenesis of AD [5].

According to the AD pathogen hypothesis, pathogens operate as triggers, in conjunction with genetic variables, in starting the accumulation and/or processing of Aβ, hyperphosphorylated tau proteins, and inflammation in the brains of AD patients [6]. HSV1 and other pathogens, such as *C. pneumoniae* and Spirochetes, are commonly found in the brains of AD patients, as these may evade the host immune response and infect the brain [7,8]. In vitro and animal studies have demonstrated that pathogens facilitate amyloid plaque formation and increased levels of hyperphosphorylated tau [9,10]. Such pathogens induce a glial inflammatory response, damaging and killing neurons directly and indirectly [11]. In the brains of AD patients, there are major inflammatory cascades [12,13], and these pathways combine to cause further neurodegeneration and disease progression.

This review examines the evidence relating to the Herpesviridae, HSV1, and CMV, and the bacterial pathogens *C. pneumoniae*, Spirochetes, periodontal pathogens, and *Helicobacter pylori*, and their relation to AD etiology. Regarding EBV and HHV6, the Herpesviridae EBV has minimal evidence of possibly contributing to AD etiology. The possible role of poisons or other environmental cofactors in the pathogenesis of AD was studied elsewhere [14]. These factors cannot be ruled out as a contributor to AD onset and progression.

## 2. AD Etiology

**Figure 1:**
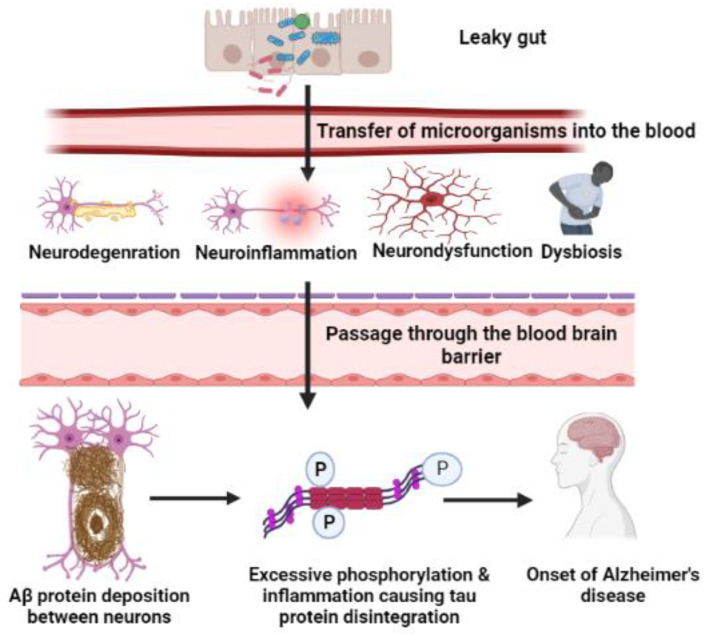
Graphical presentation depicting certain differences in gut and brain composition between healthy and unhealthy individuals. Infection with microbes inside the gut is responsible for the microbial transfer into the blood stream, and this may lead to neurodegeneration, neuroinflammation, neurodysfunction, and dysbiosis. After passing through the blood–brain barrier, a microorganism may affect APP processing, and this causes an increase in amyloid beta (Aβ) protein deposition between neurons. Inflammation among neurons and excessive phosphorylation promote tau protein disintegration, which culminates in neuronal damage and the onset of Alzheimer’s disease (AD). Dysbiosis is caused by microorganism infection, resulting in the elimination of helpful bacteria such as Firmicutes.

Identifying the contributors to AD in the human brain is a contentious topic (Figure 1); however, microorganisms appear to play a role in the progression of AD. According to evidence obtained from the postmortem examination of more than 1000 affected individuals [15], there could be a variety of factors that cause AD, and, according to a number of scientists, the primary variables in AD are the levels of Aβ and the tau protein. Aβ is produced by the APP gene, located on chromosome 2 [16], and large amounts of Aβ plaques are present not only in AD patients but also in healthy individuals. However, aggregation of Aβ and neurofibrillary tangles formed by hyperphosphorylated tau protein in the cortical and limbic areas of the human brain contribute to the pathogenesis of AD [17]. As hallmarks of AD, plaque and tangles are formed due to enzymatic excision of APP by enzymes and secretases [18,19]; these are linked to amyloidosis, inflammation, and significant synaptic brain abnormalities [20,21]. The gradual accumulation of the tau protein or Aβ aggregates or their mislocalization in unhealthy neurons leads to disproportionate proteostasis. These events impact synaptic terminal configuration and synaptic activity, eventually leading to synapse loss [22,23]. Furthermore, synaptic dysfunction is directly linked to a decline in cognitive function of the brain [24,25].

### 2.1. Role of Bacteria

Seropositive response to *Borreliabur gdorferi* (*B. burgdorferi*) was detected in both AD patients and healthy controls, but the detection rate was higher in AD patients and the onset risk of AD in seropositive IgG patients to *B. burgdorferi*. Therefore, *B. burgdorferi* may act as a risk factor for AD [26]. A previous study reported that the expression levels of Aβ and phospho-tau proteins increase after infection of *B. burgdorferi* in vitro, and *B. burgdorferi* can be detected in the brain tissues of AD patients [27]. Glial and neuronal cells have also been shown to generate amyloid precursor protein (APP) and p-tau when introduced to *B. burgdorferi* [9]. *B. burgdorferi* was originally discovered in blood and the CSF of roughly 10 infected AD and Parkinson’s patients following their brain autopsy [27,28,29]. *B. burgdorferi* was also found in studies to process Aβ precursor protein in glial and neuronal cells [3]. In the brain tissue of individuals with and without dementia, *T. denticola*, *Tannerella forsythia*, and *P. gingivalis* were observed [30]. Interestingly, the levels of LPS from *P. gingivalis* were considerably greater in the brain tissue of AD patients compared to non-AD control tissues [31]. There was also an association of the presence of Spirochetes and AD, with Spirochetes being found in over 90% of AD patient brain samples, while *B. burgdorferi* was found in roughly 25.3% using PCR and antibody detection methods. Systemically, Spirochetes and *Treponema denticola* are the most common isolated bacteria in moderate-to-severe periodontitis [32], and individuals with AD have been reported to contain periodontopathic bacteria, indicating that these microorganisms can enter the brain, possibly through peripheral nerves and the circulatory system [33]. Spirochete bacteria are also often associated with syphilitic dementia, which results in the deposition of gray matter inside the cerebral and other regions of the brain. In such instances, numerous studies have discovered an increase in spirochete colonies in the cerebral brain, primarily the treponema palladium [34]. The prevalence of oral treponemas in the trigeminal ganglia supports the notion of microbial invasion via neural pathways [35,36], and the presence of large bacterial plaques are generally considered indicators of oral bacteria in the systemic circulation. Microbes have also been linked to various neuroinflammatory and immunological conditions, and studies have shown that neuroinflammation plays a key role in AD. The brain–gut axis, also known as the brain–gut–microbiome axis, is a reversible relationship among the brain, gut, and gut bacteria [37]. 

### 2.2. Role of Viruses

Several studies have also linked HSV1 and Herpesviridae members, such as Epstein–Barr virus (EBV), Cytomegalovirus (CMV), and HHV6, to AD occurrence [38,39,40,41,42]. For HSV1/2 and EBV, such viruses are capable of latent residence in the peripheral nervous system, and they target the same parts of the central nervous system, namely, the temporoparietal cortex and hippocampus, impacted in AD and in acute cases of encephalitis (for HSV1/2 and EBV) [43]. The Herpes simplex viruses such as HSV6, HSV7, HSV8, Varicella-Zoster virus (VZV) (HSV3), EBV (HSV4), and CMV (HSV5) are neurotrophic and neuroinvasive with double-stranded DNA belonging to the Herpesviridae family [44]. Among this group, HSV1 has been demonstrated to target the trigeminal ganglia or olfactory neurons in the peripheral nervous system [45,46], and HSV1 has been detected in the brain samples, particularly for the APOE4 genotype with susceptibility to AD [43]. HSV1 incidence also includes 3.7 billion people worldwide aged 50 or older, or 67% of the population [47]. The levels of amyloidosis in the AD brain samples also correlate with levels of HSV1, implying that HSV1 is involved in the progression of AD [48]. Herpes simplex virus type 1 (HSV1) is the infection most commonly related to amyloidosis in AD patients [49,50,51,52]. Proteins from the Herpesviridae family have been found to interact with products of multiple AD susceptibility genes, namely, apolipoprotein E (APOE), phosphatidylinositol-binding clathrin assembly protein (PICALM), complement receptor 1 (CR1), and clusterin (CLU), which are associated with dementia [7,53,54]. It was found that HSV1 upregulates amyloid beta (Aβ) generation, and Herpesviridae DNA signals colocalize with amyloid plaques in AD samples [7]. The activity of Aβ with respect to the presence of neurotropic Herpesviridae virus HSV1, human herpes virus 6A (HHV6A), and human herpes virus 6B (HHV6B) was demonstrated. Herpesviridae are common human infections, with neuro-infection rates surpassing 100% in the general population [55]. Between extended periods of dormancy, HSV1 undergoes acute and episodic reactivation. HHV6A and HHV6B are also implicated in an expanding number of chronic inflammatory illnesses with foci inside and outside the CNS due to their ongoing low levels of replication [44]. Researchers have also discovered an increased abundance of Roseoloviruses HHV6 and HHV7, as well as HSV1, in the brains of AD patients [56]. Following the effects of such infections, extensive beta production with Aβ fibrils and neuroinflammation has been shown in 2D and 3D brain models of the human brain using human-induced pluripotent stem cells (hiPSCs) and HSV1 infection [45].

### 2.3. Role of Fungi

Fungal presence has also been detected via a Fungitell test for the blood samples obtained from AD patients who had Aβ deposition, leading to neurofibrillary tangles (NFTs), according to Alonso and colleagues [57]. It has also been observed that consuming antifungal medications reduces the incidence of dementia [58]. It is known that fungal infections can result in inflammatory responses, in addition to bringing vascular changes [59]. Brain samples from 11 AD patients out of a total of 29 showed strong positivity to most Candida species examined, while two more patients showed high detection of a single Candida species. *Candida famata* and *Candida albicans* are also both gut microbes, with *Candida albicans* having also been reported on vaginal surfaces [60].

### 2.4. AD and Periodontis

Periodontitis is a disorder caused by bacteria such as *Aggregatibacter* *actinomycetemcomitans*, *Fusobacterium* *nucleatum*, *Treponema* *denticola*, and *Tannerella forsythia*, which cause inflammation affecting tooth support and promoting gum damage. There is also gingivitis caused by *P. gingivalis* bacteria, which can lead to chronic systemic inflammation, as well as bone and soft-tissue degeneration in the jaw, also leading to tooth loss [33,61,62,63]. AD and periodontitis carry comparable risk factors. In plaque-induced gingivitis, half of the bacteria are Gram-positive; however, as the extent of inflammation increases, the number of Gram-negative bacteria also increases. Given subgingival dysbiosis with Porphyromonas gingivalis, the keystone pathogen of periodontal disease, shown to relate to AD occurrence, a hypothesis has been put forward in explaining the cause of AD along the lines of its two hallmark proteins for AD (Aβ fibrils and phosphorylated-tau tangles). It has been noted that advancement in age and loss of up to nine teeth are the primary risk factors for sporadic AD linked to chronic periodontitis [39]. Approximately 85% of bacteria in periodontitis are Gram-negative with *Aggregatibacter* *actinomycetemcomitans* (*Actinobacillus* *actinomycetemcomitans*), *Tannerella* *forsythensis*, *P. gingivalis*, and *T. denticola* considered the prime periodontal pathogens [64,65]. The Gram-negative bacterium, *Helicobacter pylori*, which is repressed by inhibition of GSK-3, causes inflammation and neurotoxicity by upregulating tau phosphorylation [66]. A link between periodontal disease and amyloid buildup in the brains of healthy elderly subjects was discovered using positron emission tomography (PET) imaging [65]. 

### 2.5. Other Factors Responsible for AD

Another prominent cause of AD is the plaque formation outside of the neuron as part of the AD cascade hypothesis with the aberrant formation of APP cleavage products due to the beta- and gamma-secretase cleavage of APP C-terminal residues, leading to the formation of APP83 and APP99 cleavage products. In a healthy individual, however, APP C-terminal cleavage occurs with the help of alpha and gamma-secretase, thought to facilitate immune stimulation and inflammation in the central nervous system [17].

Although it is still unclear if microorganisms such as bacteria, fungi, and viruses play a role in neurological diseases, we can claim that microbes are associated with neuro-inflammatory and immunological responses on the basis of multiple research findings. Host bacteria are primarily found as a part of human gut microbiota for digestive tract symbiosis. Periodontal bacteria, Actinobacteria, Bacteroidetes, Firmicutes, and Proteobacteria, are all examples of such gut bacteria. In addition, viruses from the Herpesviridae and Retroviridae families, particularly the HSV, have been linked to AD. As previously noted, fungi such as Candida spp., Cladosporium, *Rhodotorula* *mucilaginosa*, Malassezia sp., *Saccharomyces cerevisiae*, and Penicillium may also be implicated in neurological disease [67]. Experimental findings include viruses and bacterial genetic material found in multiple AD patients’ brains. 

Inflammation in the brain is thought to be brought about by two independent mechanisms: first, a loss of BBB in the systemic circulation, and second, agents that affect certain neuronal pathways [68,69]. An example of such agents is lipopolysaccharide (LPS) [70]. Upon binding of LPS to LPS-binding protein (LBP), LPS can signal to CD14 receptor-positive cells [71]. In these cells, LPS binds to the CD14/TLR4/MD4 receptor complex, causing the secretion of cytokines such as TNF, IL-1, IL-6, and IL-8, which in turn promote the production of prostaglandins and leukotrienes that cause inflammation and the onset of septic shock promoted by monocyte and macrophages. There is also the release of histamine when LPS binds to the CD14/TLR4/MD4 receptor complex, leading to vasodilation and stimulation of the coagulation cascade [72].

In 1998, Balin and colleagues examined patients with diverse medical histories, some of whom died from cancer, cardiac arrest, multiorgan failure, respiratory failure, systemic infection, and other severe diseases, with late-onset AD confirmed in their autopsy through histopathologic examination by a certified neuropathologist. PCR, electron, and immunoelectron microscopy of tissues for relevant AD brain structures also revealed chlamydial infection with transcriptional activity in neuropathology regions of the brains of AD patients, indicating that bacterial infection may be a significant predictor of late-onset AD. Samples were analyzed from diverse brain regions that display AD-related neuronal loss (e.g., hippocampus and temporal cortex), and a zone often mildly affected or unscathed by AD, i.e., the cerebellum. In addition, *C. pneumoniae* (Chlamydiaceae family) transcripts were detected by RT-PCR; this family of organisms also causes infections in the respiratory system and lungs [73]. Pneumonia is a general cause of death in AD patients [74]. Yeast is a strong model for basic cellular and molecular biology research, and 31% of 6000 genes are preserved between yeast and human species [75,76]. In addition, *S. cerevisiae* is mostly used as a major yeast model for AD study because it is suitable for expression studies of Aβ and tau protein [77,78].

## 3. Gut Microbiota and AD

Several gut bacteria are responsible for controlling and sustaining the host’s health [79]. Such a microbial community helps regulate neurotransmitter metabolism, form biological substances in the gut under normal physiological conditions, and maintain a healthy and balanced environment known as eubiosis [37,80]. Disruption of this ecosystem, such as via excessive antibiotic use, immune system suppression, and changes in gastrointestinal barriers, can lead to disorders including dysbiosis, which is associated with conditions such as inflammatory bowel disease (IBD), obesity, allergies, type 1 diabetes mellitus, autism, and colorectal cancer [79,81]. Microbes in the gut, such as bacteria and viruses, have been documented as pathogens responsible for various acute and chronic disorders, impacting various organs by various means from their site of infection [7,79,82].

**Figure 2:**
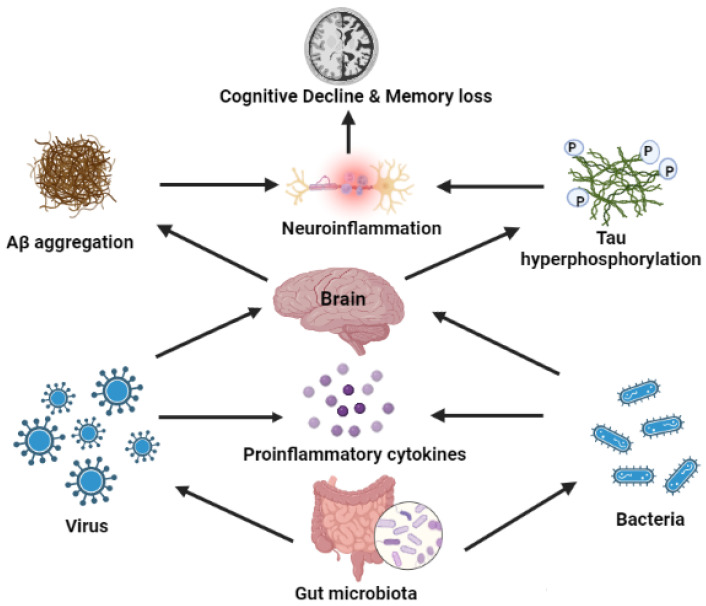
The mechanisms behind the neurological consequences of microorganisms affecting the brain. The gut microbiome imbalance affects the shortage of microbiome diversity and leads to an enhancement of intestinal permeability. Released neurotoxic agents such as proinflammatory cytokines from microorganisms can transmit to the brain through interactions between the intestine’s enteric nervous system and central nervous system. This results in the breakdown of tight junctions of the blood–brain barrier and symptoms such as Aβ aggregation (amyloid plaques and oxidative stress), neuro-inflammation, tau hyperphosphorylation, and memory loss. Lastly, it can contribute to the pathogenesis of neurological diseases like AD.

The possibility of potential therapeutic interventions increases as a pivotal role for the microbiome in the etiology of AD, and a close interrelation with the gut is found. Dysbiosis is characterized by an imbalance in the microbial population, leading to a shortage of microbiome diversity in the digestive tract or an enhanced intestinal permeability [83]. The discovered interactions between the intestine’s enteric nervous system and the central nervous system have established a link between gut bacteria and brain physiology and function [37,79]. Various biological molecules are exchanged between these two systems as they flow through the bloodstream and pass through the gut mucosa and the BBB. Neurotoxic agents such as D-lactic acid and ammonia produced by the gut bacteria can potentially cause neuronal injury [84], and the release of proinflammatory substances such as cytokines and immune activators capable of generating neuro-inflammation can start an inflammatory process in the brain [85,86]. With an imbalance in gut microbiota metabolic activities, there is also a causal component for anxiety, depression, cognitive impairment, learning, and behavioral issues seen in various neurodegenerative disorders, including AD (Figure 2) [79,84,87].

## 4. Effects of Bacterial and Viral Infection on AD

**Figure 3:**
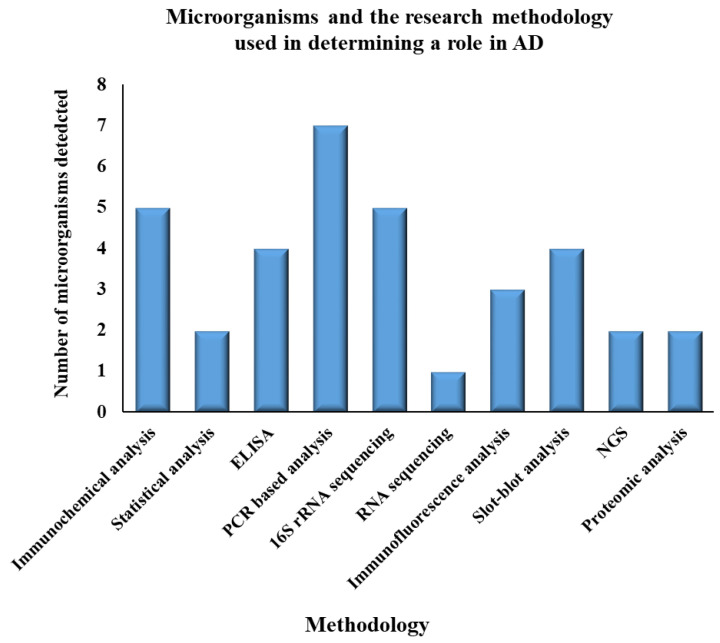
Graph depicting the methodology employed to detect the microorganism involved in causing Alzheimer’s disease. According to the data, most of the microorganisms were detected using the PCR-based analysis method, whereas very few were detected using RNA sequencing technology.

According to recent studies, gut bacteria may have a role in AD development (Table 1) [88,89,90]. Various research methodology has been utilized to identify the microorganisms associated with AD progression (Figure 3). Lactobacillus species, Bifidobacteria (Actinobacteria), Verrucomicrobia, *Spirochetes*, *Proteobacteria*, Fusobacteria, Firmicutes, and Cyanobacteria are among the bacteria that make up the gut microbial community [88], and there are signs that bacterial infection may initiate some of the degenerative processes linked to AD. Bacterial infections such as *Helicobacter pylori* [91], *B. burgdorferi*, *Chlamydia pneumonia* [92], *Escherichia coli*, *Shigella*, *Eubacterium rectale* [93], and *Bacteriodesfragilis* have all been linked to AD, with some, however, working together to reduce their infection load in AD patients. The production of neurotoxic chemicals is one route via which these bacteria might cause their harmful effects. For example, in the central nervous system, Lactobacillus species and actinobacteria may metabolize glutamate to create gamma-aminobutyric acid (GABA) [88,94]. Such elevated levels of GABA levels in the gut can impact the GABA levels in the central nervous system, and high amounts of GABA in neuronal cells are linked to memory loss, depression, and synaptic disruption [88,95].

Inflammatory activators, Aβ peptides, and other neurotoxic chemicals are also released by gut bacteria, which can impair the host immune system [57,92]. These bacteria can create endotoxins, such as lipopolysaccharides, typically present in the outer membrane of Gram-negative bacteria [96]. Gram-negative bacteria that produce LPS are abundant in the stomach, saliva, respiratory and urinary tract, skin, dental plaques, and lungs [97]. Concentrations of LPS rise following a bacterial infection or changes in the metabolic activities of gut microbiota caused by inflammation, which in turn can lead to neurodegeneration [86,97]. Regarding the functional effects of LPS on the CNS, previous research has shown that LPS causes microglial activation, memory difficulties, and overall neural damage in mice [98]. LPS also stimulates Aβ aggregation and amyloid plaque formation, which are key pathogenic processes in the progression of AD [96]. Miklossy and colleagues discovered the existence of amyloid plaques that resembled the plaques in mice following the inhalation of *C. pneumoniae*. Spirochetes were also found to cause amyloid-like symptoms in neuronal and glial cells, which might be connected to neurodegeneration [99].

Viruses, particularly the HSV and the cytomegalovirus have been linked to AD [10,100]. Inflammatory signals produced by viral infections may harm neurons, resulting in neurodegeneration [43]. For example, HSV1 has been demonstrated to cause the generation of Aβ peptide and an increase in tau phosphorylation, which can lead to Aβ peptide aggregation, the development of amyloid plaques, and neurofibrillary tangles in cell and animal models [10,101]. HSV1 was documented to cause the development of Aβ peptides (A40 and A42) in human neuroblastoma cells. HSV1 also caused hyperexcitability and increased intracellular calcium signals in rat cortical neurons, resulting in modification of the amyloid precursor processing pathway and an aberrant rise in Aβ formation [10,42]. Another example is HHV6 found in the hippocampus, frontal and temporal cortex, and hippocampus of AD patients [49]. Despite the evidence that the HHV6 virus is not directly linked to AD, investigations have revealed that it augments the harm produced by the HSV1 virus and may create brain lesions in dually infected individuals [101].

## 5. Animal Models Used to Identify a Role for Various Microorganisms in AD

### 5.1. Mouse Models

Various mouse models have been used to define the role of microorganisms in AD progression, and they are listed in Table 2. Examples of the experiments shown assayed activation changes in the brain of APOE^−/−^ mice, alterations in microbiome composition, changes in Aβ deposition, and changes in gliosis surrounding Aβ plaques. The experimental mice were either treated with antibiotics or Gram-negative oral anaerobes (*Porphyromonas gingivalis*). In another study by Brandscheid et al., changes in the fecal microbiota with age in 5XFAD mice were correlated with changes in trypsin reduction in fecal proteins and changes in human APP expressed not only in the brain but also in the gut tissue [118]. Another study with probiotic treatment in 3XTg-AD mice correlated changes in the plasma concentration of inflammatory cytokines and gut hormones with an accumulation of Aβ aggregates and brain damage [119].

### 5.2. Drosophila Models

Drosophila has been used in various studies to identify a genetic disposition for genes related to the disease of interest. Drosophila has emerged as a model system for understanding neurodegeneration pathways in various severe neurodegenerative models. These disease models are also suitable for in vivo testing of various therapeutic drug candidates. The genetic research employing these animal models has revealed new insights into neurodegenerative processes. We believe that further investigation of these animal models will further our understanding of neurodegeneration and allow for the development of novel therapeutics for severe degenerative disorders [130]. As an example, Wu et al. used transgenic Drosophila to provide evidence of the involvement of microorganisms in AD progression. In their experiments, researchers subjected Drosophila flies to Enterobacteria infection, resulting in progressive AD by increased immunological hemocyte recruitment to the brain. Interestingly, genetic hemocyte depletion also attenuated neuroinflammation and reduced neurodegeneration [128].

## 6. Potential Therapeutic Approaches in AD

### 6.1. Fecal Microbiota Transplantation

The delivery of fecal material from a healthy donor to the gastrointestinal tract of a diseased individual is known as fecal microbiota transplantation (FMT). This is an effective method of altering gut flora. Following the repeated outbreaks of severe Clostridioides difficile infection (CDI) in North America and Europe, caused partly by exposure to the Clostridioides hypervirulent NAP1/B1/027 strain, FMT has become popular in the recent decade for addressing gut flora anomalies. Cure rates of more than 90% in difficult-to-treat patients were recorded in randomized, controlled trials demonstrating the efficacy of FMT for severe CDI [131]. Improvements in a variety of extraintestinal diseases, including autism spectrum disorder (ASD), multiple sclerosis (MS), and myoclonus dystonia, have also been documented for FMT [132]. 

Several recent investigations have found a substantial correlation between gut microbiome changes and cognitive behavior [133,134]. Using 16S rRNA gene sequencing, researchers analyzed and compared variations in the makeup of the gut microbiota in AD mice models (APP, presenilin 1 (PS1), and age-matched healthy wildtype (WT) mice) [95]. Compared to the WT group, APP and PS1 mice contained considerably fewer bacteria, proteobacteria, and actinomycetes but significantly more Bacteroidetes and Trichomonas [135]. Furthermore, germ-free mice had considerably higher brain levels of Aβ after receiving fecal samples from AD animals; however, fecal transplants from WT mice had no influence on the behavioral or health outcomes of the affected mice [136].

### 6.2. Beta-Secretase

The major therapeutic molecular targets are beta-secretase (BACE1) [137,138] and gamma-secretase [139], involved in the processing of APP, and tau proteins [140], involved in the breakdown of the microtubules that keep the axonal routes together [141,142]. Beta-secretase is the most researched protein in AD (Table 2), and its protease activity causes plaque development [142]. It splits APP into A40 and A42 fragments [143], with the latter playing a key role in Familial AD. BACE1 has a cytoplasmic tail that aids in molecular maturation and intracellular trafficking, and its inhibition can slow down plaque deposition between neurons [143]. This enzyme contains an aspartate residue as an active-site moiety [144], which is also implicated in dimer and myelin sheath production outside the cell [143]. The endoplasmic reticulum forms the immature BACE1 [144], which is then processed by the Golgi apparatus to generate a mature BACE1. Three of the four possible N-glycosylation sites in BACE1 are used during the maturation of the molecule [144]. The protein has two open and closed conformations. The enzymatic activity reduces the open conformation by wrapping the active site in a tight conformation. BACE1 also has six cysteine residues that create three disulfide linkages. These linkages are necessary for BACE1 folding and enzymatic activity. The Cys330/Cys380 residues in BACE1’s active region are critical for their stability and activity. The activity of BACE1 is also directly proportional to its environmental pH, and a moderately acidic pH of 5.5 produces the highest activity levels. A free BACE1 also has an open flap conformation and is energetically stable, containing optimal electrovalent bonds [144].

### 6.3. Gamma-Secretase

Gamma-secretase cleaves CTF-beta of 99 residues, resulting in plaque-forming Aβ and CTF-gamma of 50 APP intracellular domains (AICD) plus three transmembrane domain residues [145]. Gamma secretase has an aspartyl protease activity comparable to that of bacteria’s type-4 prepilin enzyme [146], and comparable inhibitors of serine, cysteine, and aspartyl proteases can block gamma-secretase activity [147]. Gamma-secretase, however, lacks the conserved active site residues (D[TS]GS[SAT]) present in the catalytic site of nucleus-containing cell aspartyl proteases [148]. After mutation of PS1 and PS2, production of gamma-secretase increases, resulting in the development of the Aβ in AD [149]. Furthermore, the two subunits, nicastrin and anterior-pharynx defective-1, function as cofactors, forming a stable and active complex of four secretase enzymes with PS1 and PS2 [150]. Several miRNAs also boost the activity of the gamma-secretase, which results in production of Aβ; among these, MiR-9 is involved in neuronal health and synthesis of Aβ in the brain. In neurons, secretase synthesis is reduced when such complexes are inhibited, and the amyloidogenic pathways are redirected to nonamyloidogenic pathways.

### 6.4. Tau

Tau is a mature neuron’s main microtubule associated protein (MAP). The other two neuronal MAPs are MAP1 and MAP2. Interaction of MAPs with tubulin promotes its assembly into microtubules and increases the stability of the microtubule network, a well-established function of MAPs. Tau, a phosphoprotein, regulates its phosphorylation to promote microtubule assembly. In a normal adult human brain, tau protein has 2–3 moles of phosphate per mole. Tau hyperphosphorylation reduces tau’s biological activity. In AD, brain, tau is three to four times more hyperphosphorylated than in normal adult brain, and it polymerizes into paired helical filaments (PHF) admixed with straight filaments (SF), creating neurofibrillary tangles. Tau is transiently hyperphosphorylated throughout brain development, as well as during anesthesia and hypothermia, although not to the same extent as in AD brain samples. Tau has 352–441 amino acids and three domains (projection, proline-rich, and assembly) [151]. Extensive data imply that tau hyperphosphorylation is caused by a disruption in cellular signaling, specifically an imbalance in the activity of several protein kinases and phosphatases. It indicates that the Aβ plays a critical role in causing this imbalance in AD [152]. The active-site residues of the Tau protein (PDB ID: 6PXR) (Arg^57^, Ser^52^, Ser^54^, Ser^53^, Trp^100^, Asp^101^, and Glu^39^) participate in complex formation with its ligands, and this information may help in developing a tau-targeted therapeutics for AD (https://www.rcsb.org/structure/6PXR, accessed on 5 September 2022) [153].

### 6.5. APP Forms in AD

Chromosomes 1, 14, 11, 19, and 21 have the senilin, SORL1, APOE4, and APP genes responsible for depositing amyloid plaques and disassembling microtubules [154,155,156]. Over 32 different APP missense versions have been identified from 85 AD contracted families. Most of these mutations are located at the secretase cleavage sites or the APP transmembrane domain on exons 16 and 17. APP was first cloned by screening a cDNA library, encoding a 695 amino-acid protein consisting of 18 exons. The APP gene is located on chromosome 21q21, which can be alternatively spliced into several products, listed as APP695, 714, 751, 770, and 563. The expressed form of APP varies differently according to its tissue type, with three isoforms being most relevant to AD. The APP forms, APP 751 and APP 770, are expressed in both the peripheral and the central nervous systems [157,158].

### 6.6. Drugs Approved for the Treatment of AD

Studies of autosomal dominant familial AD provide the greatest evidence for the “amyloid cascade hypothesis”, a primary pathophysiologic paradigm in AD [159]. FDA-approved AD therapeutic, aducanumab, an Aβ-directed monoclonal antibody of immunoglobulin gamma 1 (IgG1) subtype, passes the blood–brain barrier (BBB) and targets the soluble oligomers and insoluble fibrils of Aβ plaques [160,161,162]. It binds to the linear epitope of the N-terminal of Aβ amino acids 3–7 [163], reduces Aβ plaques in the brain, and decreases the levels of phosphorylated tau in the CSF and medial temporal NFTs [164]. Aducanumab is given via an intravenous (IV) infusion to the patient [162]. In the last few years, various classes of inhibitors developed for beta- and gamma-secretase have reduced the accumulation of Aβ. Various compounds for beta- and gamma-secretase inhibitors have been synthesized and studied as therapeutic targets for AD. Measurements of their IC50, EC50, and Ki values (µM) are listed in Table 3 and Table 4, respectively. Compounds were synthesized by modifying the N- and C-terminal ends of beta- or gamma-secretase, and studies are ongoing for their further development. It is expected that effective candidates for AD will be reported in the future.

### 6.7. Other Therapeutic Approaches

Evidence suggests that Alzheimer’s disease is closely related to type 2 diabetes. Several studies in animal models highlighted the fact that insulin plays a major role in controlling various activities in the brain. Insulin enhances the clearance of Aβ and phosphorylates the tau protein. Glucagon-like peptide-1 has shown promising results in animal models by enhancing neuroregulation. Thus, it can be employed for treating Alzheimer’s disease [193]. Moreover, the inhibition of the production of Aβ by some enzymatic treatments is also a potential therapeutic approach in treating Alzheimer’s disease. The APP is cleaved by the γ-secretase and β-secretase enzymes that produce Aβ, which accumulates in the form of plagues and can cause Alzheimer’s disease [194]. Yu et al. stated that anti-neuroinflammatory therapy is a very effective treatment against AD. The astrocyte modulators, microbiome therapy, microglia modulator, and insulin resistance are four key strategies that comprise this anti-neuroinflammatory therapy [195]. The gut microbiome’s communication with the brain in both directions of the brain–microbiome–gut axis has received great attention with regard to the treatment of the Alzheimer’s disease. Therefore, the maintenance of GM, particularly the addition of probiotics, is a promising candidate for treating AD [196]. Krüger et al. performed a study with 161 subjects with AD and concluded that three randomized clinical trials patients who received *Bifidobacterium* and *Lactobacillus* strains had no benefits of probiotic supplementation on the cognitive functions. Probiotic supplementation did, however, improve insulin resistance, as well as very-low-density lipoprotein (VLDL) cholesterol, plasma triglyceride, and malondialdehyde levels. No RCTs evaluated the composition of the microbiota or incorporated synbiotic supplementation [197].

## 7. Microorganism-Mediated Gut Barrier Dysfunction, BBB Passage, and Activation of Chemokines/Cytokines, Leading to Breakdown of APP

### 7.1. Gut Inflammation and Dysbiosis

In the case of mucosal structure disruption, intestinal inflammatory processes drive polymorphonuclear cells to migrate to the gut mucosa from circulation or even farther to the gut lumen [198]. Calprotectin content in the feces can be used to quantify the process of intestinal inflammation indirectly. This tiny protein, an S100A8/A9 heterodimer, makes up about 60% of the neutrophil cytosol protein content and possesses antibacterial properties [199]. The S100A8 and S100A9 residues also contain amyloidogenic amino-acid sequences and may form amyloid oligomers and fibrils that mimic amyloid polypeptides such as Aβ and α-syn. Monomeric and dimeric S100A9 have been shown to promote Aβ fibrillization in vitro [200,201]. In one study, higher fecal calprotectin levels were discovered in nearly 70% of AD patients, and it is thought that calprotectin may translocate into circulation and contribute to neuroinflammation [202]. Such an intestinal supply of calprotectin might help promote amyloid fibril formation in the gut or directly in the brain [203].

Gut inflammation and dysbiosis are linked to gut barrier malfunction and increased intestinal permeability (termed a “leaky gut”). These may contribute to the neurodegenerative process [4]. The intestinal barrier is made up of the mucus layer, the intestinal epithelium, and the lamina propria [203]. When this barrier is breached, permeability increases, allowing germs and toxic chemicals to enter the circulation (a process called atopobiosis) [204,205,206]. The presence of mucin-degrading bacteria enhances gut barrier function and decreases obesity and systemic inflammation [204,207]. *Lactobacillus plantarum*, *E. coli* strain Nissle, and *Bifidobacterium infantis* are probiotic bacteria that improve the intestinal barrier by boosting the production of proteins that create tight junctions [208]. However, exotoxins, another type of bacterial substance, impair epithelial cell integrity. Changes in tight junctions have been documented for different pathogenic *E. coli* strains, *Salmonella*, *Shigella*, *Helicobacter pylori*, *Vibrio*, or *Clostridium*. For *Bacteroides fragilis*, its exotoxin damages adherence junctions by cleaving the cell adhesion molecules, the E-cadherin [208].

**Figure 4:**
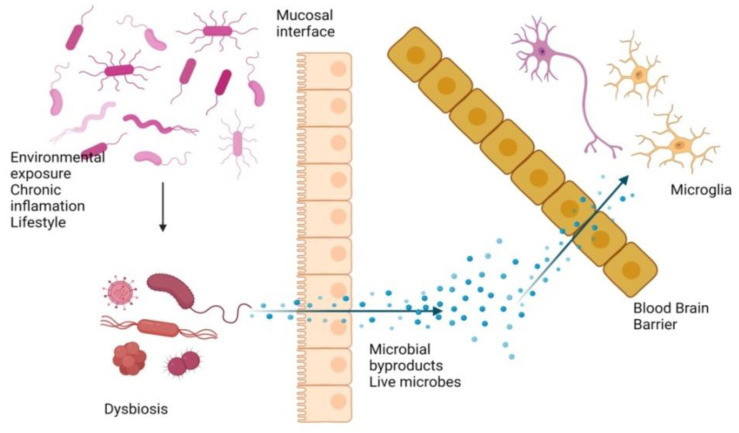
Permeation of microbial byproducts (toxins) and live microorganisms through the mucosal layer as part of the disruption in the BBB; result in disassembly, accumulation, and inflammation between neurons, which lead to AD.

Several decades of research have also demonstrated a microbiota–gut–brain axis [209] and the alteration of APP causing AD [203] and several other neurological disorders. Fungal, viral, and bacterial microorganisms bind and activate certain surface proteins on target cells; for example, some viruses bind to the C-type lectin domain family 5 member A (CLEC5A) of macrophages and activate a cytokine and chemokine storm [144]. These bioactive chemicals are specialized to break down the tight junctions present between the endothelial cells of the BBB [210]; this allows microorganisms to enter the CNS via a transcellular route through the endothelial cells [211], causing AD. IL-6, IL-7, and TNF-α are among the known inflammatory chemicals secreted by the immune cells near the BBB (Figure 4).

Alterations in the gut microbiome have been reported to affect the etiology of AD involving immunological, endocrine, and metabolic functions. Xi J. et al. suggested that bacteria-influenced or -secreted chemicals may trigger systemic inflammatory responses known as secondary metabolites and disrupt the BBB, thus promoting neurodegeneration [212]. Oral bacteria such as *P. gingivalis*, *Treponema*, and *Candida* metabolites include short-chain fatty acids (SCFAs). Such SCFAs generated by local bacteria protect Th17 cells and their function in the oral mucosa in combating Candida infection. However, abnormally low or high amounts of SCFA can be a sign of inflammation or dysbiosis [213]. Additionally, it has been demonstrated in several studies that SCFA prevents Aβ aggregation [214].

### 7.2. Amyloid Cascade Hypothesis

For the past 20 years, research has been dominated by the amyloid cascade theory, with the premise of the buildup of the amyloid-peptide in the brain as the key event in the pathophysiology of AD. Aducanumab (Biogen) is the first FDA-approved monoclonal antibody for treating AD by removing amyloid plaques [215]. Aducanumab targets Aβ and reduces the Aβ plaques in the brains of transgenic mice [160]. The clinical trials demonstrated that the brain significantly reduced Aβ plaques in a time- and dose-dependent manner. However, increased amyloid-related imaging abnormality (ARIA) was observed depending on the dose and the APOE4 genotype (Clinical Trials: NCT01397539, NCT01677572) [216]. Studies on the effectiveness and safety of aducanumab are still not enough as FDA approved it in a hurry. Therefore, aducanumab treatment will require further validation of its continued use and instructions for a prescription. Other targeted treatments based on this hypothesis are in the research phase or being tested in clinical trials. However, some treatments intended to decrease amyloid production or aggregation have failed in clinical trials. Aβ immunotherapy with bapineuzumab, tramiprosate, and solanezumab have shown failure in phase III clinical trials, as they displayed no clinical improvements for various AD biomarkers. Similarly, in phase II clinical trials, tau aggregation inhibitors and gamma-secretase inhibitors have not shown any sufficient improvements for the various markers of AD [217,218,219,220,221,222]. Therefore, it may be important to review the science underlying the amyloid cascade hypothesis on whether amyloid-directed therapeutics will prove to be lifesaving against this debilitating disease.

## 8. Conclusions

AD is linked to neurological damage and progressive synaptic dysfunction. Two major proteins, the Aβ peptide and tau, have been linked to AD and, thus, used as diagnostic markers [223,224]. Chronic AD can occur due to microorganisms, which can cause the disease after being dormant in the body. Such bacterial, fungal, and viral microorganisms may contribute to AD as they become more active with a decline in systemic immunity due to aging [51]. Periodontal pathogens and gut microorganisms such as *Aggregatibacter* *actinomycetemcomitan* (*Actinobacillus* *actinomycetemcomitans*), *Tannerella* *forsythensis*, *P. gingivalis*, *T. denticola*, and *Helicobacter pylori*, instigate inflammation and neurotoxicity by upregulating tau phosphorylation [28]. *Lactobacillus* species, *Bifidobacteria* (Actinobacteria), Verrucomicrobia, Spirochetes, Proteobacteria, Fusobacteria, Firmicutes, and Cyanobacteria, *B. burgdorferi*, *Chlamydia pneumoniae*, *E. coli*, *Shighella*, *E. rectale*, and *Bacteriodesfragilis* have all been linked to AD. Viruses, particularly HSV, HHV, and Cytomegalovirus, facilitate neurodegeneration by boosting tau phosphorylation, accumulation of Aβ peptide, senile plaques, and neurofibrillary tangles [10,54,225]. Several reports suggest the importance of microorganisms in the pathology of AD. However, since some microorganisms are also detected in healthy individuals, projecting microorganisms as a marker for AD requires detailed studies on the association and standardization between microorganisms and AD [214,226].

There are currently few therapies available for AD, which develops from dementia, and there is no known cure. The varied drug use amongst individuals is likely the most common limitation when employing bacterial quantification from the gut in AD patients. People with Alzheimer’s and dementia must typically take medications to treat the disease’s wide range of symptoms, which can significantly impact gut composition. A person may have only a specific number of particular taxa in their microbiota, which is a significant restriction in the research of the gut microbiota of AD patients. Taken together, increasing knowledge about the association of microorganisms with AD shows that they might be projected as a potential biomarker for AD in the near future.

## Figures and Tables

**Table 1:** Microorganisms and the research methodology used in determining a role in Alzheimer’s disease (AD).

S.No.	Name of Microorganisms	Role in AD	Methodology	Year	Reference
1	*Helicobacter pylori*	▪ Upregulation of Tau protein ▪ Neuroinflammation ▪ Neurotoxicity	▪ Immunochemical analysis ▪ Statistical analysis ▪ ELISA ▪ PCR based analysis	2005, 2013, 2015	[92,102,103,104]
2	Actinobacteria	Modulation of specific gene expressions	16S rRNA sequencing	2017	[105]
3	Firmicutes	Cerebral Aβ amyloidosis	16S rRNA sequencing	2017	[105,106]
4	Proteobacteria	Release of proinflammatory cytokines	16S rRNA sequencing	2017	[105]
5	*Treponema* *denticola*	Inflammation in brain	▪ 16S rDNA sequencing ▪ ELISA	2009, 2012, 2019, 2020	[107,108,109,110]
6	*Gingivitis* bacteria	▪ Alteration of tau and ubiquitin ▪ Leading to chronic inflammation	16S rDNA sequencing	2019	[109]
7	*Porphyromonas* *gingivalis*	▪ Cleavage of Tau protein ▪ Phosphorylate neuronal tau	Serological studies (ELISA)	2009	[107,111]
8	*Tannerella forsythia*	Inflammation	▪ PCR-based analysis ▪ Immunochemical analysis	2019	[31]
9	*Toxoplasma gondii*	▪ Inflammation in CNS ▪ Activation of T cells ▪ Oxidative stress	Immunochemical analysis	2015	[112]
10	Herpes simplex Virus (HSV)	▪ Neurotrophic ▪ Neuroinvasive ▪ Brain infection ▪ Encephalitis in adults ▪ Meningitis in neonates ▪ Lesions in infected individuals’ brains	▪ ELISA ▪ PCR-based analysis ▪ RNA sequencing ▪ Statistical analysis	2015, 2003, 2002, 2014, 2018	[56,101,104,113,114,115]
11	Saccharomyces cerevisiae	Oxidative stress	▪ Immunofluorescence analysis ▪ Slot-blot analysis, ▪ Proteomic analysis ▪ PCR- based analysis	2014, 2017	[58,59,116,117]
12	Malassezia species	Neuroinflammatory responsethrough T-cell activation	▪ PCR-based analysis ▪ Immunochemical analysis ▪ Immunofluorescence analysis ▪ Slot- blot analysis	2014	[58,59]
13	Candida species	Activation of NF-κB leading to proinflammatory cytokines IL-6, IL-1, and TNF	▪ Proteomic analysis ▪ PCR-based analysis ▪ Immunochemical analysis ▪ Slot- blot analysis ▪ NGS	2014, 2017	[58,59,116,117]
14	Cladosporium cryptococcus	Neuroinflammation	▪ PCR-based analysis ▪ Slot- blot analysis ▪ NGS	2014, 2017	[58,59,116,117]

**Table 2:** Mouse and Drosophila models used to identify a role for various treatments in AD.

S.No.	Name of Microorganisms	Treatment to Model	Result of Experiment	Year	Reference
1	APOE^−/−^ mice	Porphyromonas gingivalis active invasion	Activation in APOE^−/−^ mice brain	2015	[120]
2	APP/PS1 mice	Antibiotic treatment to Tg mice	▪ Changes in composition of intestinal microbiome ▪ Decrease concentration in Aβ deposition and soluble Aβ increased ▪ Reactive gliosis decreased ▪ Expansion of Lachnospiraceae ▪ Aβ deposition in aged Tg mice is reduced ▪ Plaque-localized microglia and astrocytes reduced in antibiotic-exposed mice	2016, 2017, 2018,	[121,122,123,124]
3	5XFAD mice	Composition of fecal microbiota changed along with age	▪ Enzyme trypsin in human fecal matter decreased ▪ APP expression seen in the gut tissues.	2017	[118]
4	3XTg-AD mice	Mice treated with probiotics	▪ Concentration of plasma influenced in the case of inflammatory cytokines and gut hormones ▪ Accumulation of Aβ aggregates and brain damage reduced	2017	[119]
5	AD mouse model (ICV injection of Aβ)	Bifidobacterium breve strain A1given orally	Function of hippocampus enhanced	2017	[125]
6	AD rat model (IP injection of D- galactose)	Mice treated with Lactobacillus plantarum MTCC 1325	Acetylcholine level is restored, formation of Aβ plaque attenuated	2017	[126]
7	AD rat model (intrahippocampal injection of Aβ)	Mice treated with Lactobacillus and Bifidobacterium	Learning problems and oxidative stress observed	2018	[127]
8	Transgenic flies: Drosophila	Drosophila exposed to Enterobacteria infection	▪ Neuroinflammation is reduced when hemocytes are genetically depleted ▪ Neurodegeneration reduced	2017	[128]
9	5XFAD mice	Multi-antibiotic treatment - Gentamicin (0.1251 mg/mL) - Vancomycin (0.0635 mg/mL) - Metronidazole (0.25 mg/mL) - Neomycin (0.0635 mg/mL) - Ampicillin (0.1251 mg/mL) - Kanamycin (0.3753 mg/mL) - Colistin (7,506,000 U/mL) - Cefoperazone (0.1251 mg/mL)	▪ Manipulation of the gut microbiome ▪ Mice revealed antidiabetic properties ▪ Effect on nest building quality	2021	[129]

**Table 3:** IC50, EC50, and Ki values of bioactive beta-secretase inhibitors, a therapeutic target for AD (from various studies of the last 5 years).

No.	IUPAC Names of Compounds	CID	SID	AID	IC50 (µM)	EC50 (µM)	Ki Value (µM)	Reference
1	1-N-[(2S,3S,5R)-3-amino-6-(4-fluoroani-li-no)-5-methyl-6-oxo-1-phenylhexan-2-yl] -3-N,3-N-dipropyl- benzene-1,3-dicarboxamide	5494423	103477859	332040			0.026	[165,166]
2	3-benzoyl-N-[(2S,3R)-4-(cyclopropylam-ino)-3-hydroxy-1-phenylbutan-2-yl]-5-[methyl(methylsulfonyl)amino]benzamide	5327063	103478410	332040	0.098			[165,166]
3	3-N-[(2S,3R)-4-(cyclopropylamino)-3-hydroxy-1-phenylbutan-2-yl]-5-[me-thyl(methylsulfonyl)amino]-1-N-[(1R)-1-phenylethyl]benzene-1,3-dicarboxamide	5287532	103492819	332040	0.015			[165,166]
4	[3-[2-(5-aminopentylamino)-2-oxoethoxy]-5-[[(1R)-1-(4-fluorophenyl)ethyl]carbamoyl]phenyl] phenylmethanesulfonate	448772	103511935	332040	1.4			[165,166]
5	1-carbazol-9-yl-3-[4-(3-carbazol-9-yl-2-hydroxypropyl)piperazin-1-yl]propan-2-ol	2729022	103564170	332040	7			[165,166]
6	1-[4-(3-carbazol-9-yl-2-hydroxypropyl)piperazin-1-yl]-3-(3,6-dichlorocarbazol-9-yl)propan-2-ol	2802372	103564215	332040	5			[165,166]
7	1-(3-acetylphenyl)-3-(3-ethylsulfanyl-1,2,4-thiadiazol-5-yl)urea	11198061	103459736	239369			28.8	[167,168]
8	5-[[3,5-bis(trifluoromethyl)phenyl]car-bam-oylamino]-2-(dimethylamino)-N-(3-morpholin-4-ylpropyl)benzamide	4243074	103459765	239369			8.1	[167,168]
9	5-[[3,5-bis(trifluoromethyl)phenyl]car-bam-oylamino]-2-(dimethylamino)-N-(3-methoxypropyl)benzamide	5040262	103459791	239369			9.4	[167,168]
10	5-[(3-chlorophenyl)carbamoylamino]-2-(dimethylamino)-N-(3-ethoxypropyl)benzamide	3948694	103460050	239369			9.8	[167,168]
11	1-(3-acetylphenyl)-3-(3-ethylsulfanyl-1,2,4-thiadiazol-5-yl)urea	11198061	103459736	240366		0.0114		[167,169]
12	5-[[3,5-bis(trifluoromethyl)phenyl]car-bam-oylamino]-2-(dimethylamino)-N-(3-methoxypropyl)benzamide	5040262	103459791	240366		27		[167,169]
13	5-[(3-chlorophenyl)carbamoylamino]-2-(dimethylamino)-N-(3-ethoxypropyl)benzamide	3948694	103460050	240366		0.0233		[167,169]
14	5-[[3,5-bis(trifluoromethyl)phenyl]car-bam-oylamino]-2-(dimethylamino)-N-(3-morpholin-4-ylpropyl)benzamide	4243074	103459765	240368		0.0035		[167,170]
15	tert-butyl N-[1-[[(4S)-8-[[1-(benzyl-amino)-3-methyl-1-oxobutan-2-yl]amino]-5-hydroxy-2,7-dimethyl-8-oxooctan-4-yl]amino]-4-methylsulfanyl-1-oxobutan-2-yl]carbamate	44305543	103256754	238396			5.808	[171,172]
16	tert-butyl N-[(2S)-1-[[1-[[(4S)-8-[[1-(benzylamino)-3-methyl-1-oxobutan-2-yl]amino]-5-hydroxy-2,7-dimethyl-8-oxooctan-4-yl]amino]-4-methylsulfonyl-1-oxobutan-2-yl]amino]-3-methyl-1-oxobutan-2-yl]carbamate	44305867	103257369	238396			0.008	[171,172]
17	tert-butyl N-[(2S)-1-[[1-[[(4S)-8-[[1-(benzylamino)-3-methyl-1-oxobutan-2-yl]amino]-5-hydroxy-2,7-dimethyl-8-oxooctan-4-yl]amino]-4-methylsulfanyl-1-oxobutan-2-yl]amino]-3-methyl-1-oxobutan-2-yl]carbamate	44305868	103257370	238396			0.0025	[171,172]
18	tert-butyl N-[(2S)-1-[[1-[[(4S)-8-[[1-(benzylamino)-3-methyl-1-oxobutan-2-yl]amino]-5-hydroxy-2,7-dimethyl-8-oxooctan-4-yl]amino]-3-methylsulfonyl-1-oxopropan-2-yl]amino]-3-methyl-1-oxobutan-2-yl]carbamate	44305869	103257371	238396			0.0094	[171,172]
19	tert-butyl N-[(2S)-1-[[4-amino-1-[[(4S)-8-[[1-(benzylamino)-3-methyl-1-oxobutan-2-yl]amino]-5-hydroxy-2,7-dimethyl-8-oxooctan-4-yl]amino]-1,4-dioxobutan-2-yl]amino]-3-methyl-1-oxobutan-2-yl]carbamate	44305870	103257372	238396			0.0059	[171,172]
20	tert-butyl N-[(2S)-1-[[4-amino-1-[[(4S)-8-[[1-(benzylamino)-1-oxopropan-2-yl]amino]-5-hydroxy-2,7-dimethyl-8-oxooctan-4-yl]amino]-1,4-dioxobutan-2-yl]amino]-3-methyl-1-oxobutan-2-yl]carbamate	44305918	103257473	238396			0.0614	[171,172]
21	tert-butyl N-[(2S)-1-[[1-[[(4S)-8-[[1-(benzylamino)-3-methyl-1-oxobutan-2-yl]amino]-5-hydroxy-2,7-dimethyl-8-oxooctan-4-yl]amino]-3-methylsulfanyl-1-oxopropan-2-yl]amino]-3-methyl-1-oxobutan-2-yl]carbamate	44305946	103257506	238396			0.0501	[171,172]
22	(4S)-4-amino-5-[[(2S)-1-[[(2S)-4-amino-1-[[(4S,5S,7R)-8-[[(2S)-1-[[(2S)-4-carboxy-1-[[(1S)-1-carboxy-2-phenylethyl]amino]-1-oxobutan-2-yl]amino]-1-oxopropan-2-yl]amino]-5-hydroxy-2,7-dimethyl-8-oxooctan-4-yl]amino]-1,4-dioxobutan-2-yl]amino]-3-methyl-1-oxobutan-2-yl]amino]-5-oxopentanoic acid	445649	103282992	238396			0.0016	[171,172]
23	tert-butyl N-[(3S)-1-amino-5-[[(4S)-8-[[1-(benzylamino)-1-oxopropan-2-yl]amino]-5-hydroxy-2,7-dimethyl-8-oxooctan-4-yl]amino]-1,4-dioxopentan-3-yl]carbamate	44394717	103451640	238396			22.423	[171,172]
24	tert-butyl N-[(3S)-1-amino-5-[[(4S)-8-[[1-(benzylamino)-3-methyl-1-oxobutan-2-yl]amino]-5-hydroxy-2,7-dimethyl-8-oxooctan-4-yl]amino]-1,4-dioxopentan-3-yl]carbamate	44394718	103451641	238396			3.134	[171,172]
25	tert-butyl N-[(2R)-4-[[(4S)-8-[[1-(benzylamino)-3-methyl-1-oxobutan-2-yl]amino]-5-hydroxy-2,7-dimethyl-8-oxooctan-4-yl]amino]-1-methylsulfonyl-3-oxobutan-2-yl]carbamate	44394719	103451642	238396			1.129	[171,172]
26	(2R,5S)-5-[[(2S)-2-[[(2R,4S,5S)-5-[[(2S)-2-[[(2S)-2-[[(2S)-2-amino-4-carboxybutanoyl]amino]-4-methylpentanoyl]amino]-3-carboxypropanoyl]amino]-4-hydroxy-2,7-dimethyloctanoyl]amino]-3-methylbutanoyl]amino]-2-benzyl-4-oxooctanedioic acid	44394812	103451744	238396			0.00032	[171,172]
27	1-N-[(2S,3S,5R)-5-(benzylcarbamoyl)-1-(3,5-difluorophenyl)-3-hydroxyheptan-2-yl]-3-N,3-N-dipropylbenzene-1,3-dicarboxamide	10121628	103222903	240791	1.4			[171,173]
28	1-N-[(2S,3S,5R)-1-(3,5-difluorophenyl)-5-[[(3S,5R)-3,5-dimethoxycyclohexyl]carbamoyl]-3-hydroxyheptan-2-yl]-3-N,3-N-dipropylbenzene-1,3-dicarboxamide	11354322	103222904	240791	6.6			[171,173]
29	5-[[(2R,4S,5S)-6-(3,5-difluorophenyl)-5-[[3-(dipropylcarbamoyl)benzoyl]amino]-2-ethyl-4-hydroxyhexanoyl]amino]pentanoic acid	10282555	103222935	240791	0.4			[171,173]
30	3-[[(2R,4S,5S)-6-(3,5-difluorophenyl)-5-[[3-(dipropylcarbamoyl)benzoyl]amino]-2-ethyl-4-hydroxyhexanoyl]amino]propanoic acid	10167359		240791	2.8	-		[171,173]
31	4-[[[(2R,4S,5S)-6-(3,5-difluorophenyl)-5-[[3-(dipropylcarbamoyl)benzoyl]amino]-2-ethyl-4-hydroxyhexanoyl]amino]methyl]cyclohexane-1-carboxylic acid	9809811	103223159	240791	0.05			[171,173]
32	[(2S,3S)-2-(3,4-dihydroxyphenyl)-5,7-dihydroxy-3,4-dihydro-2H-chromen-3-yl] 3,4,5-trihydroxybenzoate	65056	103359341	44249	4.5			[95,96]
33	(2R,3S)-2-(3,4,5-trihydroxyphenyl)-3,4-dihydro-2H-chromene-3,5,7-triol	65084	103359373	44249	2.5			[174,175]
34	[(2R,3S)-5,7-dihydroxy-2-(3,4,5-trihydroxyphenyl)-3,4-dihydro-2H-chromen-3-yl] 3,4,5-trihydroxybenzoate	5276890	103359448	44249	1.8			[174,175]
35	(2S,3S)-2-(3,4-dihydroxyphenyl)-3,4-dihydro-2H-chromene-3,5,7-triol	182232	103359466	44249	28			[174,175]
36	(2S,3S)-2-(3,4-dihydroxyphenyl)-3,4-dihydro-2H-chromene-3,5,7-triol	182232	103359466	44249	23			[174,175]
37	(2S,3S)-2-(3,4,5-trihydroxyphenyl)-3,4-dihydro-2H-chromene-3,5,7-triol	10425234	103359508	44249	2.4			[174,175]
38	[(2S,3R)-2-(3,4-dihydroxyphenyl)-5,7-dihydroxy-3,4-dihydro-2H-chromen-3-yl] 3,4,5-trihydroxybenzoate	6419835	103359509	44249	6			[174,175]
39	[(2S,3S)-5,7-dihydroxy-2-(3,4,5-trihydroxyphenyl)-3,4-dihydro-2H-chromen-3-yl] 3,4,5-trihydroxybenzoate	2824823	103359580	44249	1.6			[174,175]
40	(2S,3R)-2-(3,4-dihydroxyphenyl)-3,4-dihydro-2H-chromene-3,5,7-triol	73160	103474775	44249	30			[174,175]
41	(2R,3S)-2-(3,4-dihydroxyphenyl)-3,4-dihydro-2H-chromene-3,5,7-triol	9064	123094711	44249	35			[174,175]
42	[(2S,3S)-2-(3,4-dihydroxyphenyl)-5,7-dihydroxy-3,4-dihydro-2H-chromen-3-yl] 3,4,5-trihydroxybenzoate	65056	103359341	44250			5.3	[174,176]
43	[(2R,3S)-5,7-dihydroxy-2-(3,4,5-trihydroxyphenyl)-3,4-dihydro-2H-chromen-3-yl] 3,4,5-trihydroxybenzoate	5276890	103359448	44250			0.17	[174,176]
44	[(2S,3S)-5,7-dihydroxy-2-(3,4,5-trihydroxyphenyl)-3,4-dihydro-2H-chromen-3-yl] 3,4,5-trihydroxybenzoate	2824823	103359580	44250			0.21	[174,176]
45	5-[[3,5-bis(trifluoromethyl)phenyl]carbamoylamino]-2-(dimethyl- amino)-N-(3-morpholin-4-ylpropyl)benzamide	4243074	103459765	242654	0.0578			[167,177]
46	5-[(3-chlorophenyl)carbamoylamino]-2-(dimethylamino)-N-(3-ethoxypropyl)benzamide	3948694	103460050	242654	0.097			[167,177]
47	(4R)-4-[[(2R)-2-[[(2R)-2-[[(3S)-4-[(4-butoxybenzoyl)amino]-3-hydroxy-6-methylheptanoyl]amino]-3-methylbutanoyl]amino]propanoyl]amino]-5-[[(1R)-1-carboxy-2-phenylethyl]amino]-5-oxopentanoic acid	44296588	103236975	44238	27			[178,179]
48	methyl 4-[[(2R)-2-[[(3S)-3-hydroxy-6-methyl-4-[[(2S)-2-[[(2S)-3-methyl-2-[(2-methylpropan-2-yl)oxycarbonylamino]butanoyl]amino]-4-methylsulfanylbutanoyl]amino]heptanoyl]amino]-3-methylbutanoyl]amino]butanoate	44296483	103236754	44238	47			[178,179]
49	3-[[(2R)-2-[[(3S)-3-hydroxy-6-methyl-4-[[(2S)-2-[[(2S)-3-methyl-2-[(2-methylpropan-2-yl)oxycarbonylamino]butanoyl]amino]-4-methylsulfanylbutanoyl]amino]heptanoyl]amino]-3-methylbutanoyl]amino]propanoic acid	44296454	103236696	44238	30			[178,179]
50	4-[[[(2R)-2-[[(3S)-3-hydroxy-6-methyl-4-[[(2S)-2-[[(2S)-3-methyl-2-[(2-methylpropan-2-yl)oxycarbonylamino]butanoyl]amino]-4-methylsulfanylbutanoyl]amino]heptanoyl]amino]-3-methylbutanoyl]amino]methyl]benzoic acid	44296453	103236695	44238	4			[178,179]

**Table 4:** IC50, EC50, and Ki values of bioactive gamma secretase inhibitors, a therapeutic target for AD (from various studies of the last 5 years).

**No.**	**IUPAC Names of Compounds**	**CID**	**SID**	**AID**	**IC50** **(µM)**	**EC50** **(µM)**	**PMID**	**Reference**
1	(2R)-2-[4-[(R)-(4-fluorophenyl)-(4-methylpiperidin-1-yl)methyl]-3-[4-(trifluoromethyl)phenyl]phenyl]propanoic acid	53493001	163326581	1678948		0.15	33479693	[180,181]
2	(2R)-2-[4-[(R)-(4-fluorophenyl)-(4-methylpiperidin-1-yl)methyl]-3-[4-(trifluoromethyl)phenyl]phenyl]propanoic acid	53493001	163326581	1678949	0.17		33479693	[180,182]
3	5-[4-[8-(3,4-dichlorophenyl)-5,6,7,8-tetrahydro-[1,2,4]triazolo[4,3-a]pyridin-3-yl]-2-methoxyphenyl]-2-methyl-1,3-oxazole	66608062	318390383	1678953	0.24		33479693	[180,183]
4	6-chloro-3′-[3-methoxy-4-(2-methyl-1,3-oxazol-5-yl)phenyl]-1-(2,2,2-trifluoroethyl)spiro[5H-4,1-benzoxazepine-3,8′-6,7-dihydro-5H-[1,2,4]triazolo[4,3-a]pyridine]-2-one	57521199	336903002	1678953	0.03		33479693	[180,183]
5	3′-[3-methoxy-4-(2-methyl-1,3-oxazol-5-yl)phenyl]-1-(2,2,2-trifluoroethyl)-6-(trifluoromethyl)spiro[5H-4,1-benzoxazepine-3,8′-6,7-dihydro-5H-[1,2,4]triazolo[4,3-a]pyridine]-2-one	127049947	336903439	1678953	0.031		33479693	[180,183]
6	2-[(8R)-3-[3-methoxy-4-(2-methyl-1,3-oxazol-5-yl)phenyl]-8-(3,4,5-trifluorophenoxy)-6,7-dihydro-5H-[1,2,4]triazolo[4,3-a]pyridin-8-yl]propan-2-ol	56837196	336903440	1678953	0.026		33479693	[180,183]
7	2-[8-(3-chloro-4-fluorophenoxy)-3-[3-methoxy-4-(2-methyl-1,3-oxazol-5-yl)phenyl]-6,7-dihydro-5H-[1,2,4]triazolo[4,3-a]pyridin-8-yl]propan-2-ol	66603958	336905149	1678953	0.038		33479693	[180,183]
8	8-[(3,4-difluorophenyl)methyl]-3-[3-methoxy-4-(2-methyl-1,3-oxazol-5-yl)phenyl]-N-(2,2,2-trifluoroethyl)-6,7-dihydro-5H-[1,2,4]triazolo[4,3-a]pyridine-8-carboxamide	66604236	336906847	1678953	0.075		33479693	[180,183]
9	8-(4-fluoro-2-methylphenyl)-N-(1-methylindazol-5-yl)-[1,2,4]triazolo[1,5-a]pyridin-2-amine	142607770	404669659	1678954	1.2		33479693	[180,184]
10	8-(4-fluoro-2-methylphenyl)-N-(1-methyl-4,5,6,7-tetrahydroindazol-5-yl)-[1,2,4]triazolo[1,5-a]pyridin-2-amine	142607900	404671937	1678954	0.38		33479693	[180,184]
11	N-[3,3-difluoro-1-(3-methyl-1,2,4-thiadiazol-5-yl)piperidin-4-yl]-8-(2,3,4-trifluorophenyl)-[1,2,4]triazolo[1,5-a]pyridin-2-amine	58397894	461518428	1678954	0.143		33479693	[180,184]
12	(3E)-1-[(1S)-1-(4-fluorophenyl)ethyl]-3-[[3-methoxy-4-(4-methylimidazol-1-yl)phenyl]methylidene]piperidin-2-one	11560787	104244932	1678955	0.093		33479693	[180,185]
13	(2R)-2-[4-[(R)-(4-fluorophenyl)-(4-methylpiperidin-1-yl)methyl]-3-[4-(trifluoromethyl)phenyl]phenyl]propanoic acid	53493001	163326581	1678967		0.146	33479693	[180,186]
14	(2R)-2-[4-[(R)-(4-fluorophenyl)-(4-methylpiperidin-1-yl)methyl]-3-[4-(trifluoromethyl)phenyl]phenyl]propanoic acid	53493001	163326581	1678968	0.064		33479693	[180,187]
15	(2R)-2-[3-chloro-4-(2,2,2-trifluoroethoxy)-5-[4-(trifluoromethyl)phenyl]phenyl]-3-cyclobutylpropanoic acid	71623049	318386441	1678971	0.067		33479693	[180,188]
16	(2S)-2-[[(2S)-2-(3,5-difluorophenyl)-2-hydroxyacetyl]amino]-N-[(7S)-5-methyl-6-oxo-7H-benzo[d][1]benzazepin-7-yl]propanamide	10435235	103537689	359781		0.000119	17573346	[189,190]
17	1-(2-fluorophenyl)-3-(4-methoxy-2-nitrophenyl)urea	2992227	103719617	452857	10		19853461	[191,192]
18	1-(3,4-dimethylphenyl)-3-(2-hydroxy-4-nitrophenyl)urea	46226838	103719560	452857	15		19853461	[191,192]
19	1-(2-hydroxy-4-nitrophenyl)-3-(2,3,4-trifluorophenyl)urea	46226843	103719569	452857	8		19853461	[191,192]
20	1-(3,5-dimethylphenyl)-3-(2-hydroxy-4-nitrophenyl)urea	46226853	103719584	452857	2		19853461	[191,192]
21	1-(2-ethyl-6-methylphenyl)-3-(2-hydroxy-4-nitrophenyl)urea	46226857	103719593	452857	7		19853461	[191,192]
22	1-(2-ethyl-6-propan-2-ylphenyl)-3-(2-hydroxy-4-nitrophenyl)urea	46226858	103719594	452857	3		19853461	[191,192]
23	1-(2-fluorophenyl)-3-(2-methoxy-4-nitrophenyl)urea	310495	103719605	452857	5		19853461	[191,192]
24	1-(3-fluorophenyl)-3-(2-methoxy-4-nitrophenyl)urea	4580964	103719606	452857	12		19853461	[191,192]
25	1-(2-hydroxy-4-nitrophenyl)-3-(2,4,5-trifluorophenyl)urea	46226866	103719607	452857	10		19853461	[191,192]
26	1-(2-hydroxy-4-nitrophenyl)-3-(2-methylphenyl)urea	22011319	103719608	452857	2		19853461	[191,192]
27	1-(2,5-dimethylphenyl)-3-(2-hydroxy-4-nitrophenyl)urea	46226837	103719559	452857	10		19853461	[191,192]
28	1-(2-ethylphenyl)-3-(2-hydroxy-4-nitrophenyl)urea	21184795	103719618	452857	0.09		19853461	[191,192]
29	1-(2-hydroxy-4-nitrophenyl)-3-(2-propylphenyl)urea	46196416	103719619	452857	0.1		19853461	[191,192]
30	1-(2-hydroxy-4-nitrophenyl)-3-[2-(2-methylpropyl)phenyl]urea	46226874	103719620	452857	1		19853461	[191,192]
31	1-(3-ethylphenyl)-3-(2-hydroxy-4-nitrophenyl)urea	46226884	103719634	452857	4		19853461	[191,192]
32	1-(4-ethylphenyl)-3-(2-hydroxy-4-nitrophenyl)urea	46226885	103719635	452857	3		19853461	[190,191]
33	1-(2-hydroxy-4-nitrophenyl)-3-(2-methyl-6-propan-2-ylphenyl)urea	46226886	103719636	452857	5		19853461	[190,191]
34	1-(2,4-dibromophenyl)-3-(2-hydroxy-4-nitrophenyl)urea	22011336	103719649	452857	0.8		19853461	[191,192]
35	1-(2,4-dichlorophenyl)-3-(2-hydroxy-4-nitrophenyl)urea	22011310	103719650	452857	0.3		19853461	[191,192]
36	1-(2-hydroxy-4-nitrophenyl)-3-(2,3,4-trichlorophenyl)urea	46226814	103719527	452857	3		19853461	[191,192]
37	1-(2-bromophenyl)-3-(2-hydroxy-4-nitrophenyl)urea	3854666	103547644	452857	0.5		19853461	[191,192]
38	1-[2,6-di(propan-2-yl)phenyl]-3-(2-hydroxy-4-nitrophenyl)urea	46226772	103719467	452857	8		19853461	[191,192]
39	1-(2,4-difluorophenyl)-3-(2-hydroxy-4-nitrophenyl)urea	46226786	103719483	452857	15		19853461	[191,192]
40	1-(2,5-dichlorophenyl)-3-(2-hydroxy-4-nitrophenyl)urea	4592749	103719484	452857	0.5		19853461	[191,192]
41	1-(3,4-dichlorophenyl)-3-(2-hydroxy-4-nitrophenyl)urea	46226804	103719508	452857	0.7		19853461	[191,192]
42	1-(3,4-difluorophenyl)-3-(2-hydroxy-4-nitrophenyl)urea	46226805	103719509	452857	8		19853461	[191,192]
43	1-(2-hydroxy-4-nitrophenyl)-3-(4-propylphenyl)urea	21184782	103719510	452857	4		19853461	[191,192]
44	1-(2-fluorophenyl)-3-(2-hydroxy-4-nitrophenyl)urea	21184605	103719525	452857	10		19853461	[191,192]
45	1-(2-chlorophenyl)-3-(2-hydroxy-4-nitrophenyl)urea	9883044	103719526	452857	0.6		19853461	[191,192]
46	1-(2-hydroxy-4-nitrophenyl)-3-phenylurea	3618472	103195215	452857	12		19853461	[191,192]
47	1-(2-hydroxy-4-nitrophenyl)-3-(2,4,5-trichlorophenyl)urea	46226815	103719528	452857	3		19853461	[191,192]
48	1-[2-ethyl-6-(2-methylpropyl)phenyl]-3-(2-hydroxy-4-nitrophenyl)urea	46226816	103719529	452857	15		19853461	[191,192]
49	1-(2-fluorophenyl)-3-(4-nitrophenyl)urea	2985799	2985799	452857	30		19853461	[191,192]
50	1-(2-hydroxy-4-nitrophenyl)-3-(2,4,6-trifluorophenyl)urea	46226828	103719547	452857	15		19853461	[191,192]

## Data Availability

Not applicable.
